# Antiproliferative and Antimetastatic Effects of Praeruptorin C on Human Non–Small Cell Lung Cancer through Inactivating ERK/CTSD Signalling Pathways

**DOI:** 10.3390/molecules25071625

**Published:** 2020-04-01

**Authors:** Chien-Ming Liu, Huan-Ting Shen, Yi-An Lin, Yung-Luen Yu, Yong-Syuan Chen, Chung-Jung Liu, Yi-Hsien Hsieh

**Affiliations:** 1 Department of Pulmonary Medicine, Taichung Tzu Chi Hospital, Buddhist Tzu Chi Medical Foundation, Taichung 40427, Taiwan; lpaladin@tzuchi.com.tw (C.-M.L.); ryenhat@tzuchi.com.tw (H.-T.S.); 2 Institute of Biochemistry, Microbiology, and Immunology, Chung Shan Medical, Taichung 40201, Taiwan; kevin810647@gmail.com; 3 Department of Medical Laboratory and Biotechnology, Chung Shan Medical University, Taichung 40201, Taiwan; anyalin8831829@gmail.com; 4 Graduate Institute of Biomedical Sciences, China Medical University, Taichung 40402, Taiwan; ylyu@mail.cmu.edu.tw; 5 Center for Molecular Medicine, China Medical University Hospital, Taichung 40402, Taiwan; 6 Department of Biotechnology, Asia University, Taichung 40402, Taiwan; 7 Division of Gastroenterology, Department of Internal Medicine, Kaohsiung Medical University Hospital, Kaohsiung Medical University, Kaohsiung 80708, Taiwan; 8 Regenetative Medicine and Cell Therapy Research Center, Kaohsiung Medical University, Kaohsiung 80708, Taiwan; 9 Institute of Medicine, Chung Shan Medical University, Taichung 40201, Taiwan; 10 Department of Biochemistry, School of Medicine, Chung Shan Medical University, Taichung 40201, Taiwan; 11 Clinical Laboratory, Chung Shan Medical University Hospital, Taichung 40201, Taiwan

**Keywords:** Praeruptorin C, non-small cell lung cancer, proliferation, invasion, cathepsin D

## Abstract

Praeruptorin C (PC) reportedly has beneficial effects in terms of antiinflammation, antihypertension, and antiplatelet aggregation, and it potentially has anticancer activity. However, the effect of PC on human non–small cell lung cancer (NSCLC) is largely unknown. Compared with the effects of praeruptorin A and praeruptorin B, we observed that PC significantly suppressed cell proliferation, colony formation, wound closure, and migration and invasion of NSCLC cells. It induced cell cycle arrest in the G0/G1 phase, downregulated cyclin D1 protein, and upregulated p21 protein. PC also significantly reduced the expression of cathepsin D (CTSD). In addition, the phosphorylation/activation of the ERK1/2 signalling pathway was significantly suppressed in PC-treated NSCLC cells. Cotreatment with PC and U0126 synergistically inhibited CTSD expression, cell migration, and cell invasion, which suggests that the ERK1/2 signalling pathway is involved in the downregulation of CTSD expression and invasion activity of NSCLC cells by PC. These findings are the first to demonstrate the inhibitory effects of PC in NSCLC progression. Therefore, PC may represent a novel strategy for treating NSCLC.

## 1. Introduction

Lung cancer is the most commonly diagnosed cancer and the leading cause of cancer-related deaths globally because of its poor prognosis [1]. According to data from the GLOBOCAN database, 2.09 million new cases of lung cancer are reported yearly (11.6% of all cancer cases), resulting in 1.76 million deaths (18.4% of all cancer-related deaths) worldwide [2]. Non–small cell lung cancer (NSCLC) is the most common type of lung cancer, comprising 85% of all lung cancers. NSCLC can be divided into subtypes, such as lung adenocarcinoma (40%), lung squamous cell carcinoma (25–30%), and large cell carcinoma (10–15%) [3,4]. Metastasis and drug resistance are the main causes of death in lung cancer patients [5]. Approximately 66% of patients exhibit metastatic lesions when they are first diagnosed with lung cancer [6]. Therefore, inhibition of metastasis is a critical topic in cancer research. New strategies for the treatment of lung cancer should be explored.

Because metastasis is such a concern in patients with lung cancer, many cancer treatments have focused on the prevention of metastasis. Metastasis is a complex process that involves the destruction of extracellular matrix (ECM) components, an increase in cancer cell invasion from the primary tumour site and their suspension in the circulatory system, and, finally, growth at a target organ [7,8]. Migration and invasion of cancer cells and the degradation of the ECM exacerbate the development of malignant tumours. However, cancer cells must undergo several changes for distant metastasis to occur. First, cancer cells gain the ability to migrate and invade surrounding tissues. Second, cancer cells destroy intercellular relationships. Third, the ECM is lysed or destroyed. Finally, the ability of cancer cells to adhere to the ECM is increased [9]. Cathepsin D (CTSD) plays a decisive role in bone metastasis in lung cancer by degrading basement membrane components and the ECM [10,11]. Mitogen-activated protein kinase (MAPK) superfamily members are closely associated with increased migration, invasion, proliferation, survival, and apoptosis, thus playing diverse and crucial roles in lung cancer progression [12,13].

Natural plant compounds have been investigated for potential efficacy in the treatment of various cancers. Certain phytochemicals in medicinal herbs reportedly possess antitumorigenic activity. The synergistic effects of herbal medicines and natural foods might improve the effectiveness of conventional antitumor agents or might even be suitable replacements for conventional chemotherapeutic drugs [14,15]. Praeruptorin C (PC) is a bioactive compound derived from the dried roots of *Peucedanum praeruptorum* Dunn and is used as an antioxidant and a calcium antagonist to treat diseases. PC has efficacy in lowering blood pressure and dilating coronary arteries [16], anti-hypertension [17], anti-inflammation [18], and antiplatelet aggregation properties. Furthermore, it exhibits neuroprotective abilities [19] and has potential anticancer activity [20]. However, the effects of PC on the proliferation and metastasis of NSCLC cells and the molecular mechanisms involved are still unknown. In the present study, we investigated whether PC treatment is sufficient to downregulate cell survival and suppress migration abd invasion. We also identified the precise molecular mechanisms in NSCLC cells. Our findings demonstrated that PC treatment inhibits cell proliferation, invasive motility, and CTSD expression by suppressing the ERK1/2 signalling pathway. Therefore, PC might serve as a therapeutic agent to limit cancer progression by inhibiting cell growth and invasive motility in NSCLC. 

## 2. Results

### 2.1. Effect of PC on Cell Viability and Cytotoxicity in NSCLC Cells 

**Figure 1:**
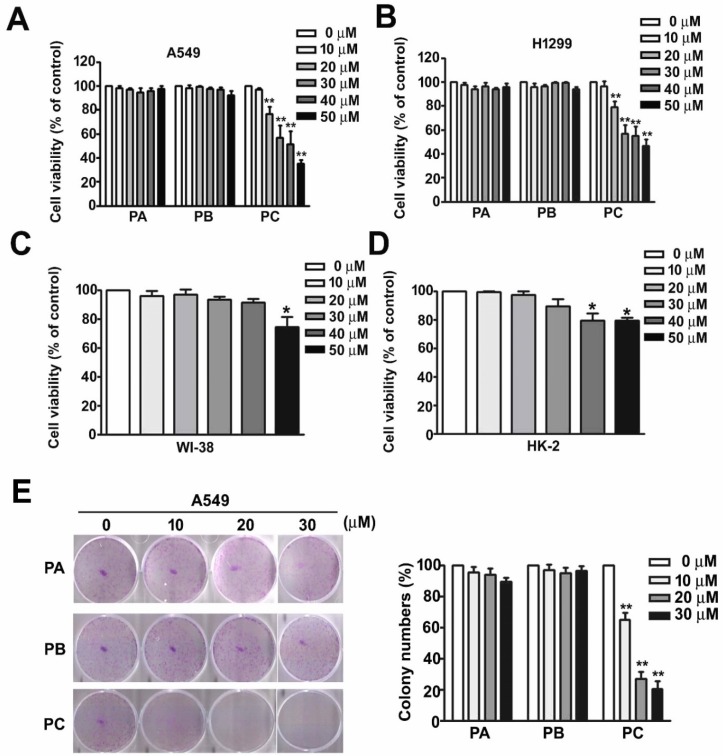
Effect of PC on cell viability and cytotoxicity in NSCLC cells. (**A**) A549 cells (human lung adenocarcinoma cell line), (**B**) H1299 cells (human lung adenocarcinoma cell line), (**C**) WI-38 cells (human lung fibroblast cell line), and (**D**) HK-2 cells (human PTC line derived from normal kidney) were treated with various concentrations (0, 10, 20, 30, 40, and 50 μM) of PA, PB, or PC for 24 h and then measured using MTT assay. (**E**) Colony formation of A549 cells treated with PC (0, 10, 20, and 30 μM) for 24 h were measured. * *p* < 0.05; ** *p* < 0.01 versus control (line 1), (Mean ± SE, *n* = 3).

We compared the effects of praeruptorin A (PA), praeruptorin B (PB), and PC on cell viability and cytotoxicity in two human lung cancer cell lines, A549 and H1299. These cells were treated with various concentrations (0, 10, 20, 30, 40, and 50 μM) of PA, PB, and PC for 24 h, followed by a MTT assay. We observed a significant decrease in cell viability in A549 (IC_50_ = 33.5 μM ± 7.5) and H1299 cells (IC_50_ = 30.7 μM ± 8.4) treated with PC, but the same effect was not observed with PA and PB treatment (Figure 1A,B). We further measured the concentration at whih cytotoxicity side effects appear in two normal cell lines, WI-38 cells (human lung fibroblast cell line) and HK-2 cells (human proximal tubular cell [PTC] line derived from a healthy kidney). PC (50 μM) treatment reduced cell viability in WI-38 cells, and PC (40 and 50 μM) treatment caused cell cytotoxicity in HK-2 cells (Figure 1C,D). Therefore, we used PC in concentrations below 30 μM to execute the subsequent experiments and studies. Colony formation was measured in A549 cells treated with PC (0, 10, 20, and 30 μM) for 24 h to confirm the effect of PC in reducing cell viability (Figure 1E). The results indicated that PC significantly inhibits NSCLC cell growth. 

### 2.2. Effect of PC on Cell Cycle Arrest in NSCLC Cells

**Figure 2:**
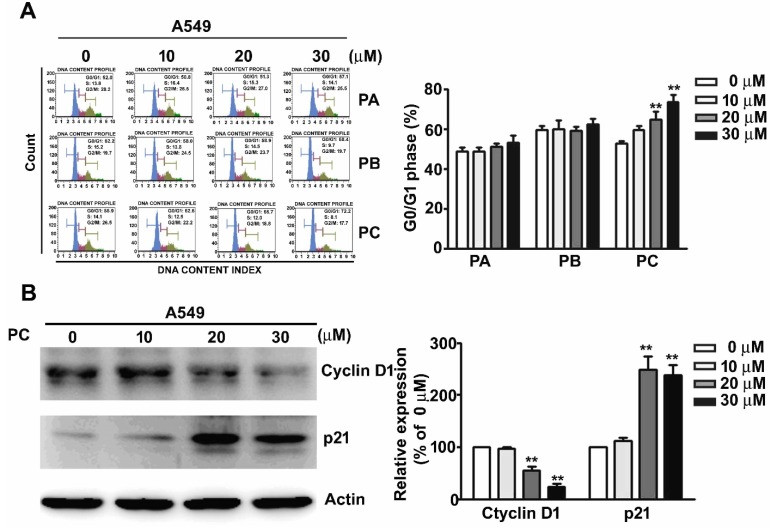
Effect of PC on cell cycle arrest in NSCLC cells. (**A**) Cell cycle and apoptosis of human A549 lung cancer cells treated with various concentrations (0, 10, 20, and 30 μM) of PA, PB, and PC were measured using flow cytometry. (**B**) Cell cycle regulation proteins, cyclin D1 and p21, were further detected to confirm the effect of PC on A549 cells. ** *p* < 0.01 versus control (line 1), (Mean ± SE, *n* = 3).

Human A549 lung cancer cells were treated with various concentrations (0, 10, 20, and 30 μM) of PA, PB, and PC for 24 h, followed by flow cytometry assay. PC treatment (20 and 30 μM) significantly induced cell arrest at the G0/G1 phase but PA and PB treatment did not (Figure 2A). Immunoblotting assay was performed to further confirm the effect of PC in the regulation of the cell cycle and induction of apoptosis by measuring cell cycle regulation proteins cyclin D1 and p21. The results indicated that PC significantly affects the induction of cell cycle arrest at the G0/G1 phase and apoptosis in these NSCLC cells (Figure 2B). 

### 2.3. PC Inhibits Cell Migration, Invasion and CTSD Expression in NSCLC Cells

**Figure 3:**
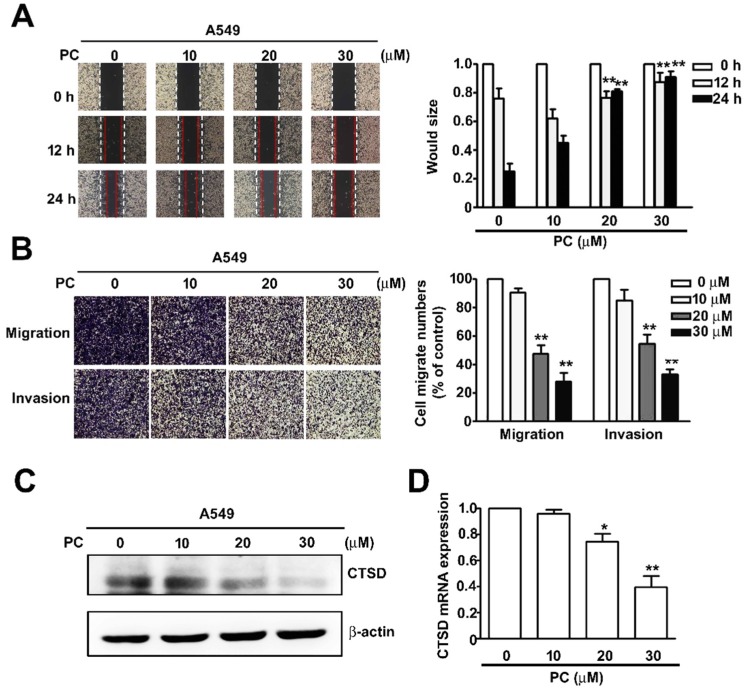
Inhibitory effect of PC on cell migration, invasion, and CTSD expression in NSCLC cells. (**A**,**B**) Human A549 lung cancer cells were treated with various concentrations of PC (0, 10, 20, and 30 μM) for 12 and 24 h, and subsequently their capacity for cell migration and invasion was measured. (**C**,**D**) Human A549 lung cancer cells were treated with PC (0, 10, 20, and 30 μM) for 24 h. Cells were then harvested for detection of CTSD gene and protein levels by RT-qPCR and immunoblotting assay. * *p* < 0.05; ** *p* < 0.01 versus control (line 1), (Mean ± SE, *n* = 3).

To identify the effect of PC on cellular migration and invasion activity in NSCLC cells, we treated human A549 cells with various concentrations of PC (0, 10, 20, and 30 μM) for 12 or 24 h and executed wound healing assay (Figure 3A) and migration and invasion assay (Figure 3B). We found that PC significantly inhibited cellular migration and invasion activity in A549 cells in a dose-dependent manner.

CTSD has been shown to be instrumental in lung cancer cell migration and invasion. To identify the effect of PC on the gene and protein expression of CTSD in NSCLC cells, we treated human A549 lung cancer cells with various concentrations (0, 10, 20, and 30 μM) of PC for 24 h, followed by immunoblotting and RT-qPCR assays. The results revealed that gene and protein levels of CTSD in human A549 cells were significantly reduced in a dose-dependent manner when exposed to PC (10, 20, and 30 μM) for 24 h (Figure 3C,D).

### 2.4. PC inhibits the Activation of the ERK1/2 Pathway in NSCLC Cells 

**Figure 4:**
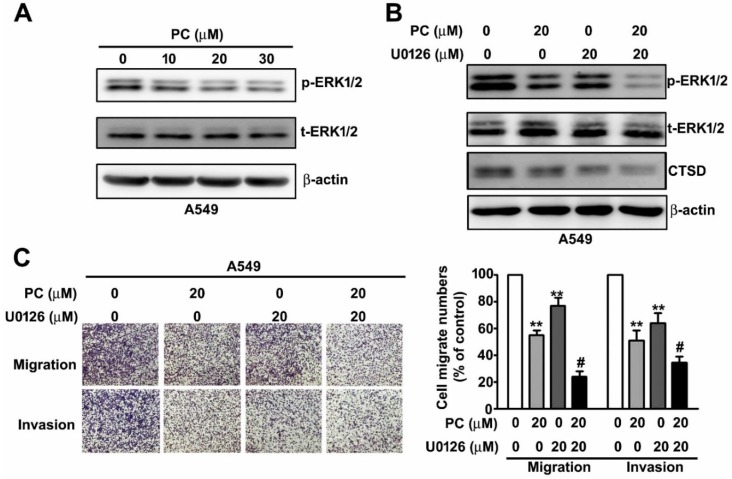
Synergistic inhibitory effect of PC and U0126 on cell migration and invasion by downregulation of the EKR1/2 signalling pathway. (**A**,**B**) Human A549 lung cancer cells were treated with PC in the presence or absence of U0126 (a specific MEK1/2 inhibitor) and then harvested for an immunoblotting assay to observe the activation of the EKR1/2 signalling pathway and CTSD protein expression. (**C**) Human A549 lung cancer cells were treated with various concentrations of PC (0 and 20 μM) and/or U0126 (0 and 20 μM) for 24 h, and subsequently their capacity for cell migration and invasion was measured. ** *p* < 0.01 versus control (line 1); #, *p* < 0.05 versus PC treatment (line 2). (Mean ± SE, *n* = 3).

To investigate which a signal transduction pathway were involved in the downregulation of migration and invasion activity in NSCLC cells after PC treatment, we treated human A549 cells with PC (0, 10, 20, and 30 μM). A549 cells were then harvested for immunoblotting assay to observe the phosphorylation/activation of the signalling pathways. We found that PC significantly reduced the phosphorylation/activation of the ERK1/2 signalling pathway (Figure 4A).

### 2.5. ERK1/2 Activation is Involved in the PC-Induced Suppression of Cell Migration and Invasion and CTSD Expression in NSCLC Cells

To examine the effect of PC and U0126 (a specific MEK1/2 inhibitor) on cellular migration and invasion activity in NSCLC cells, we subjected human A549 cells to treatments of PC (0 and 20 μM) and/or U0126 (0 and 20 μM) for 24 h. We found that individual treatments of PC (20 μM) or U0126 (20 μM) significantly inhibited CTSD expression, cellular migration, and invasion in A549 cells. Cotreatment of PC (20 μM) or U0126 (20 μM) revealed had a greater inhibitory effect on CTSD expression and invasive motility in human A549 lung cancer cells (Figure 4B,C). The results suggested that the ERK1/2-CTSD signalling pathway was involved in the suppression of migration and invasion in NSCLC cells by PC.

## 3. Discussion

Lung cancer has a low survival rate and remains the leading cause of global cancer-related deaths. Therefore, novel approaches must be urgently explored for lung cancer treatment and the prevention of its recurrence and metastasis. Many plant-derived compounds have potential bioactivities that counteract cancer development. In this study, we observed the following results. (i) According to viability, cytotoxicity, and colony formation assays, PC treatment revealed significant cell cytotoxicity and reduced cell survival in NSCLC cells. (ii) PC treatment led to a significant induction in cell cycle arrest at G0/G1 phase and apoptosis in these NSCLC cells. However, PA and PB did not influence the induction of cytotoxic effects, cell cycle arrest, or apoptosis. (iii) PC significantly suppressed cell invasive motility activity in NSCLC cells. (iv) PC significantly downregulated the expression of CTSD gene and protein levels in a dose-dependent manner. (v) PC substantially inhibited the activation of the ERK1/2 signalling pathway. (vi) Cotreatment of PC and U0126 synergistically downregulated the invasive motility activity and CTSD expression of NSCLC cells. These results demonstrate that PC downregulates migration, invasion, and CTSD expression by blocking the ERK1/2 signalling pathway in NSCLC cells.

ECM remodelling and cellular migration and invasion contribute to the distant metastasis of cancer cells through multifactorial biological processes. Cathepsin D (CTSD), an aspartyl endoproteinase, is involved in various physiological processes and signalling pathways. Studies have reported increased overexpression and hypersecretion of CTSD in many cancer types. Enhanced CTSD expression is considered an indicator of malignancy in serous ovarian cancer [20,21]. A significant overexpression of CTSD in the omental lesion of serous ovarian carcinoma was observed, and it is believed to be associated with poor disease-specific survival [22]. Higher expression of CTSD has also been correlated with an increased risk of clinical metastasis and short survival of patients with breast cancer [23,24,25]. CTSD overexpression promotes breast cancer cell migration, invasion, and metastasis by upregulating the expression of intercellular cell adhesion molecule-1 [26]. CTSD enhances the proliferation and invasiveness of NSCLC cells [27] CTSD was validated in a clinical study and could acts as a biomarker for osteosarcomas, pulmonary metastasis, and bone malignancies [28]. CTSD also has a pivotal role in the regulation of angiogenesis, metastasis, invasion, apoptosis, and cell proliferation; therefore, it is considered a potential therapeutic target for natural products in cancer chemoprevention [29]. In this study, we observed that PC inhibits gene and protein expressions of CTSD and suppresses cell migration and invasion in NSCLC cells. 

The molecular mechanisms underlying the regulation of cancer progress have been explored with the goal of identifying the complete network of signalling pathways and developing effective approaches against cancers. MAPKs include three major subfamilies, namely ERKs, JNKs, and p38 MAPK, that contribute to the control of cancer development and progression [30,31,32,33]. Huaier Granule extract suppresses proliferation and metastasis in lung cancer cells by downregulating the MTDH, JAK2/STAT3, and MAPK signalling pathways [34]. MAPK inhibitors, particularly JNK, enhance the cell death effects in H_2_O_2_-treated lung cancer cells by increasing superoxide anion and glutathione depletion [35]. The EGFR/MAPK pathway mediates aldolase A–promoted cyclin D1 expression and proliferation and G1/S transition in NSCLC [36]. Activation of the MAPK pathway by the long noncoding RNA TUC338 promotes the invasion of lung cancer [37]. The FGFR1–ERK1/2–SOX2 axis promotes cell proliferation, epithelial–mesenchymal transition (EMT), and metastasis in FGFR1-amplified lung cancer [38]. PEAK1 overexpression contributes to EMT and tumour metastasis by activating ERK1/2 and JAK2 signalling in lung cancer [39]. ERK1/2-upregulated expression of PUMA contributes to propofol-inhibited cell survival and propofol-induced cell apoptosis in NSCLC [40]. The results of the aforementioned studies suggest that targeting MAPK activation is a potential therapeutic strategy against the development and progression of human lung cancer. In this study, we observed that PC treatment significantly inhibited activation of the ERK1/2 signalling pathway. Furthermore, cotreatment of PC and U0126 synergistically suppressed migration, invasion, and CTSD expression in NSCLC cells.

The bioactivity of plant-derived compounds has shown promise as anticancer agents for increasing patients’ survival rate. Angular pyranocoumarin extracted from *P. praeruptoruom* reportedly inhibits proliferation and induces apoptosis in human U266 myeloma cells by upregulating caspase-8 and caspase-3 proteins and downregulating phospho-ERK and phospho-AKT proteins and hTERT mRNA [41]. Prenylated coumarins, the ethanol extracts of *P. praeruptorum*, exhibit in vitro cytotoxic activity against various types of human cancer cells [42]. Pyranocoumarins from the root extracts of *P. praeruptorum* Dunn, reportedly reduce nitric oxide production and inhibit the multidrug-resistant protein-mediated efflux of drugs [23]. PA has antiproliferative and cytotoxic effects and promotes the inhibitory effects of doxorubincin in human SGC7901 gastric cancer cells [43]. PA inhibits human cervical cancer cell growth and invasion by suppressing MMP-2 expression and ERK1/2 pathway activation [32]. In this study, we investigated the anticancer properties of PA, PB, and PC in NSCLC cells. PC significantly decreased cell growth by inducing cell cytotoxicity, cell cycle arrest in the G0/G1 phase, and apoptosis in NSCLC cells in comparison with PA and PB treatments. PC exhibited specific inhibitory effects in human NSCLC cells through the downregulation of cyclin D1 protein and upregulation of p21 protein. Furthermore, PC might suppress cell migration and invasion by downregulating CTSD expression and ERK1/2 pathway activation. Previously reports have been showed that ectopic expression of CTSD in CTSD-deficient fibroblasts stimulates 3D outgrowth that is associated with a significant increase in fibroblast proliferation, survival, motility, and invasive capacity via activation of ERK1/2 pathway. Furthermore, CTSD could act as a key paracrine communicator between cancer and stromal cells, independently of its catalytic activity [44]. Depletion of CTSD gene expression by RNA interference decreases proliferative response and invasive potential in human breast cancer cell through suppressing the activation of EKR1/2 pathway [45]. Epithelial ovarian cancer(EOC)-secreted CTSD acts as an extracellular ligand and induces proliferation and migration in human omental microvascular endothelial cells (HOMECs) through activation of ERK1/2 and PI3K/AKT pathways [46]. Based on our observation, we found that PC cotreated with U0126 does not affect cell viability in A549 and H1299 cells. Therefore, antiproliferative effect of PC in NSCLC cells is not involved in ERK1/2-CTSD axis, it may be participated in other signaling pathways, which we will continue to focus on these important issues of future study. 

## 4. Materials and Methods

### 4.1. Reagents 

PA, PB, and PC were purchased from ChemFaces (Wuhan, China). The stock solutions of PA, PB, and PC were prepared at a concentration of 100 mM in dimethyl sulfoxide and stored at −80 °C. Crystal violet solution, MTT and U0126 were purchased from Sigma (St. Louis, MO, USA). Cyclin D1, p21, p-ERK1/2, ERK1/2, CTSD, and β-actin antibodies were purchased from Santa Cruz Biotechnology (Santa Cruz, CA, USA). Horseradish peroxidase-conjugated antimouse, antigoat, and antirabbit secondary antibodies were also obtained from Santa Cruz Biotechnology. All stock solutions were wrapped in foil and maintained at −20 °C 

### 4.2. Cell Culture

A549 (human lung adenocarcinoma cell line), H1299 (human lung adenocarcinoma cell line), WI-38 (human lung fibroblast cell line) and HK-2 (human PTC line derived from a healthy kidney) cell lines were obtained from American Type Culture Collection (Manassas, VA, USA) and cultured in either Dulbecco’s modified Eagle’s medium (DMEM; for A549 and WI38), minimum essential medium (for H1299), or DMEM: Nutrient Mixture F-12 (DMEM/F-12; for HK2) supplemented with 10% fetal bovine serum, 2 mM glutamine, 100 U/mL penicillin, and 100 µg/mL streptomycin. All cell cultures were maintained at 37 °C in a humidified atmosphere of 5% CO_2_.

### 4.3. Cell Viability Assay

To determine the effect of PA, PB, and PC on cell viability, cells were treated with different concentrations of PA, PB, or PC (0, 10, 20, 30, 40, and 50 μM) for 24 h, and were subjected to MTT assay (Sigma). The absorbance of blue formazan crystals was measured at 570 nm using an enzyme-linked immunosorbent assay plate reader. The quantity of formazan was directly proportional to the number of viable cells in the culture medium. The cell viability of cells was determined according to the absorbance corrected to a background reading. 

### 4.4. Colony Formation Assay

Cells were seeded onto 6-well plates for 7 days. Colonies with more than 100 cells were stained with 0.5% crystal violet solution for 30 min at room temperature. Three independent experiments were performed.

### 4.5. Cell Cycle Distribution by Flow Cytometric Analysis 

Cells were centrifuged at 800 rpm at 4 °C for 5 min, washed with ice-cold phosphate-buffered saline (PBS), and stained with Muse Annexin V and dead cell reagent. The DNA content was examined using Muse Cell Analyzer flow cytometry and data were analysed using the Muse Cell Analyzer (EMD Millipore, Billerica, MA, USA). Cells were gated to exclude cell debris, doublets, and clumps. Apoptotic cells with hypodiploid DNA content were detected in the sub-G1 region.

### 4.6. Immunoblotting 

To isolate total proteins, cells were washed with cold PBS and resuspended in lysis buffer (50 mM Tris, pH 7.5, 0.5 M NaCl, 1.0 mM ethylenediaminetetraacetic acid, 10% glycerol, 1 mM β-mercaptoethanol, and 1% Nonidet P-40) with mixtures of proteinase inhibitors and phosphatase inhibitors (Roche Molecular Biochemicals, Pleasanton, CA, USA). After incubation for 30 min on ice, the supernatant was collected by centrifugation at 12,000 g for 15 min at 4 °C, and the protein concentration was determined using the Bradford method. Samples containing equal amounts of total protein (20 μg) were separated by 10–12% SDS-PAGE and transferred onto PVDF membranes (Life Technologies, Carlsbad, CA, USA). The membranes were blocked with a 5% nonfat dry milk in blocking buffer for at least 2 h at room temperature and then incubated with primary antibodies in the aforementioned solution on an orbital shaker at 4 °C overnight. Subsequently, the membranes were incubated with horseradish peroxidase–linked secondary antibodies (anti-rabbit, anti-mouse, or anti-goat IgG). Antibody-bound protein bands were detected using highly sensitive Immobilon Western Chemiluminescent HRP Substrate (Millipore), and photographed using the Luminescent Image Analyzer LAS-4000mini (GE Healthcare, Pittsburgh, PA, USA).

### 4.7. Cell Motility Determined by Wound Healing Assay 

A549 cells (4 × 10^5^ cells/well) were seeded onto a 6-well plate and grown overnight to 90% confluence. After removing the medium, we scratched the cell monolayer with a 200-μL pipette tip to create a wound. The cells were then washed twice with PBS to remove floating cells and fresh medium (containing 2 μg/mL of mitomycin c) was added. Cells migrating from the leading edge of the wound were photographed at 0, 12, and 24 h.

### 4.8. Migration and Invasion Assay 

A549 cells were treated with different concentrations of PC for 24 h by using 48-well modified Boyden chambers containing membrane filter inserts with 8-μm pores (Corning Incorporated Life Sciences, Tewksbury, MA, USA). These filter inserts were coated with Matrigel (BD Biosciences, Billerica, MA, USA) prior to the invasion assay. The lower compartment was filled with DMEM containing 10% FCS. Cells were placed in the upper part of the chamber, which contained serum-free medium, and incubated for 16–24 h. Cell migration and invasion were determined by counting cells that migrated to the lower side of the filter at 100× magnification, respectively. Four microscopic fields were counted for each filter, and each sample was assayed in triplicate.

### 4.9. Reverse Transcription and Real-Time PCR Assay 

Total RNA was isolated from cultured cells by homogenisation in TRIzol reagent (Invitrogen, Carlsbad, CA, USA) in preparation for RNA extraction. Standard RT and real-time PCR protocols were used. For RT, the samples were incubated at 25 °C for 10 min, and real-time PCR was initiated with a hot start (10 min at 95 °C, 1 cycle). Samples were then subjected to 40 cycles at 95 °C for 15 s and 60 °C for 1 min. Data were analysed using the StepOne real-time PCR system (Applied Biosystems, Foster City, CA, USA). The primers were as follows: CTSD forward primer 5′-GCAAACT GCTGGACATCGCTTG-3′, CTSD reverse primer 5′-GCCATAGTGGATGTCAAACGAGG-3′, glyceraldehyde 3-phosphate dehydrogenase (GAPDH) forward primer 5′-CATCATCCCTGCCT CTACTG-3′, and GAPDH reverse primer 5′-GCCTGCTTCACCACCTTC-3′ (Mission Biotech, Taipei, Taiwan). Relative gene expression was obtained after normalisation with endogenous GAPDH and determination of the difference in threshold cycle between treated and untreated cells using the 2−ΔΔCt method.

### 4.10. Statistical Analysis 

Results are presented as means ± standard errors (SEs), and statistical comparisons were conducted using Student’s t test by GraphPad Prism 5 (GraphPad Software, San Diego, CA, USA). Each experiment was performed at least three times. Significance was defined as a *p* value of <0.05 or <0.01.

## 5. Conclusions

Our results indicate that PC has specific anticancer abilities. It suppresses cell growth, migration, and invasion by downregulating the activation of the MEK1/2–ERK1/2 pathway and CTSD expression in NSCLC cells. To our knowledge, this study is the first to illuminate the effects and molecular mechanisms underlying the anticancer chemotherapeutic potential of PC for clinical use in combination treatments against NSCLC cells.
